# Using Stem Cells to Grow Artificial Tissue for Peripheral Nerve Repair

**DOI:** 10.1155/2016/7502178

**Published:** 2016-04-26

**Authors:** Kulraj Singh Bhangra, Francesca Busuttil, James B. Phillips, Ahad A. Rahim

**Affiliations:** ^1^Department of Biomaterials and Tissue Engineering, UCL Eastman Dental Institute, University College London, 256 Gray's Inn Road, London WC1X 8LD, UK; ^2^Department of Pharmacology, UCL School of Pharmacy, University College London, 29–39 Brunswick Square, London WC1N 1AX, UK

## Abstract

Peripheral nerve injury continues to pose a clinical hurdle despite its frequency and advances in treatment. Unlike the central nervous system, neurons of the peripheral nervous system have a greater ability to regenerate. However, due to a number of confounding factors, this is often both incomplete and inadequate. The lack of supportive Schwann cells or their inability to maintain a regenerative phenotype is a major factor. Advances in nervous system tissue engineering technology have led to efforts to build Schwann cell scaffolds to overcome this and enhance the regenerative capacity of neurons following injury. Stem cells that can differentiate along a neural lineage represent an essential resource and starting material for this process. In this review, we discuss the different stem cell types that are showing promise for nervous system tissue engineering in the context of peripheral nerve injury. We also discuss some of the biological, practical, ethical, and commercial considerations in using these different stem cells for future clinical application.

## 1. Introduction

Despite advances in microsurgical techniques and a progressive understanding of pathophysiological mechanisms, peripheral nerve repair continues to be a major clinical challenge. Peripheral nerve injury (PNI) is often accompanied by loss of sensation, partial or complete apraxia, chronic pain, and occasionally permanent disability. Causes of peripheral nerve damage include conditions such as diabetes [1], Guillain-Barré syndrome [2], and cancer [3] along with iatrogenic injuries [4], but PNI prevails in the context of trauma [5]. Estimates vary, but approximately 300,000 cases of traumatic PNI present annually in Europe alone and in the United States PNI accounts for approximately 3% of all trauma cases and 5% if plexus and root avulsions are included [6, 7].

Peripheral nerves can regenerate to some extent and this ability is mainly attributable to intrinsic growth capacity of peripheral neurons and the ability of Schwann cells to provide a supportive growth environment [8]. Following a nerve transection injury, denervated Schwann cells in the distal part of the nerve adopt a regenerative phenotype and provide support to regenerating axons from the proximal stump. However, the degree of reinnervation is dependent on many factors such as the severity of injury, interstump gap length, alignment of nerve stumps, anatomical location of injury, delay before surgical intervention, and type of repair procedure applied [9]. In the case of chronic denervation, distal Schwann cells can lose their regenerative capacity, which can lead to incomplete regeneration [10, 11].

The clinical gold standard repair strategy for repairing large gaps in transected peripheral nerves is the nerve autograft. This offers a Schwann cell-rich autologous material to bridge the interstump gap and serves to guide regenerating axons. This method is not ideal because of donor site morbidity, the requirement for additional surgery, and limited donor tissue availability. The limitations of autografting have led to the search for alternative therapies. In particular, the use of tissue engineering to construct artificial tissue that mimics the nerve autograft provides a potentially innovative solution for peripheral nerve repair. Various authors have reviewed natural and synthetic materials for nerve tissue engineering [12–15] so the aim of this review is to explore the cellular components that could be used in an engineered tissue to encourage nerve regeneration. Since the use of allogeneic Schwann cells requires a source of nerve tissue, it is affected by the same factors that limit the autograft. This has resulted in the development of a range of approaches that use stem cells as a source of therapeutic material.

The ability of stem cells to self-renew and to differentiate towards a desired lineage makes them a popular choice as the starting point for cell therapies. Nevertheless, there are issues regarding host immune response after administration, oncogenic properties that give rise to teratomas or teratocarcinomas, in addition to various ethical concerns [16, 17]. This review discusses recent studies in which stem cells have been used as sources of therapeutic cells to construct artificial peripheral nerve tissue. It also considers the practicalities associated with different sources of therapeutic cells in terms of biological and commercial feasibility for translation to the clinic.

## 2. Preclinical Studies Using Stem Cells for Peripheral Nerve Repair

The inclusion criteria for the studies in Table 1 included (1)* in vivo* experimental study in animals or humans, (2) use of a nerve conduit or graft as a scaffold for stem cell delivery, and (3) studies within the last 5 years (2010–2015). The exclusion criteria included (1)* in vitro* experimental studies, (2) use of injection as a mode of delivery of the stem cells, and (3) models of crush injuries, that is, absence of a gap between the proximal and distal stumps of the injured nerve.

## 3. Sources of Stem Cells Used in Nerve Tissue Engineering

### 3.1. Embryonic Stem Cells (ESCs)

In 1998, Thomson et al. [18] described the isolation of a pluripotent cell line from the human blastocyst. The use of ESCs to treat CNS disorders is well-documented [19–21], while it seems their potential to treat PNS injuries remains largely unexplored. Due to their pluripotency, differentiation along a specific neural lineage is challenging [22]. A study by Ziegler et al. [23] generated neurospheres from hESC by coculture with stromal cells, grown under conditions supportive of Schwann cell differentiation. After 8 weeks, hESCs differentiated into cells with morphological and molecular features characteristic of Schwann cells and associated with neurites from chick, rat, and human origin* in vitro*. Various other studies have shown that ESC can differentiate along a neural lineage [24–27].

Cui et al. [28] injected mouse ESCs into a sciatic nerve in a rat axotomy model (after the sciatic nerve was transected, the surrounding epineurium was resutured). ESCs were neurally induced and were transplanted 1 hour after removal of a 10 mm segment of nerve. Three months following axotomy, the ESCs survived and Fluoro-Gold (FG) retrograde staining along with electrophysiology showed better regeneration than controls.

Several groups have reported that ESCs share behavioural characteristics similar to cancer cells and express markers that are also found in many human and mouse cancer models [29, 30]. Undifferentiated ESCs have been known to form teratomas or teratocarcinomas [31]. In addition, there are risks of immunogenicity and various ethical barriers presented by this stem cell source [16].

### 3.2. Bone Marrow Stem Cells (BMSCs)

Several* in vitro* studies have reported that BMSCs can be induced to differentiate into neural lineages including neurons, astrocytes, oligodendrocytes, and Schwann-like cells [32]. Experimental studies in rats [33, 34], rabbits [35], dogs [36, 37], and primates [38, 39] have investigated the effectiveness of these cells in improving functional outcomes following peripheral nerve repair. Wang et al. [40] suggested that rat BMSCs can positively influence the regeneration of peripheral nerves not only through the direct release of neurotrophic factors, but also through indirect modulation of the behaviour of Schwann cells.

Caddick et al. [41] demonstrated that rat BMSCs can be differentiated into cells that are Schwann cell-like in both phenotype and function. Following differentiation with all-trans-retinoic acid (ATRA), platelet derived growth factor (PDGF), basic fibroblast growth factor (BFGF), and forskolin, BMSCs underwent morphological changes to resemble cultured Schwann cells and stained positive for the Schwann cell markers S100, P75, and glial fibrillary acidic protein (GFAP). The differentiated BMSCs were also found to enhance neurite outgrowth in coculture with sensory neurons to a level equivalent or superior to that produced by Schwann cells. Further reports indicating that differentiated BMSCs can mimic the functions of Schwann cells in culture have since been published [34, 42, 43].

Keilhoff et al. [44] showed that rat BMSCs can be differentiated* in vitro* into Schwann-like cells using a combination of cytokines. The myelinating capacity of the transdifferentiated cells was studied in coculture with PC12 cells and by grafting into an autologous muscle conduit bridging a 2 cm gap in a rat model.* In vitro*, transdifferentiated BMSCs were able to myelinate PC12 cells after 14 days while,* in vivo*, they increased the numbers of newly myelinated fibres after 3 weeks.

Autologous transdifferentiated BMSCs were transplanted into a monkey model of median nerve injury using a polymer tube containing a collagen sponge [39]. This study showed improvements in behaviour, electrophysiology, and histology for up to one year. Further studies have shown that the addition of BMSCs to conduits and acellular grafts results in superior outcomes when compared with empty channels [37, 38, 45, 46]. Furthermore, the nerve regeneration capacity of BMSCs can be dose dependent, as indicated by Raheja et al. [47], who reported better improvements in rats with a sciatic nerve transection treated with a high dose of cells compared to those treated with a low dose and controls.

### 3.3. Skeletal Muscle Derived Stem Cells (SMDSCs)

Tamaki et al. [48] reported that skeletal muscle interstitium contained multipotent stem cells. It was further established that these were able to differentiate into mesodermal cells (including skeletal muscle cells and endothelial cells) and ectodermal cells such as Schwann cells and perineurial cells* in vivo* [49]. In addition to multipotent differentiation, SMDSCs are characterized by sustained self-renewal and long-term proliferation [50]. Another potentially favourable characteristic of these cells is their survival capability under conditions of oxidative and hypoxic stresses [51].

In another study, Tamaki et al. [52] utilised a nerve crush injury model in mice to examine the differentiation capacity and contributions of undifferentiated murine SMDSCs in peripheral nerve regeneration. After 4 weeks, engrafted SMDSCs differentiated into Schwann cell-like cells which myelinated the regenerated axons. Additionally, the SMDSCs were reported to form perineurial/endoneurial cells and associated blood vessels composed of donor-derived endothelial cells, pericytes, and fibroblasts. Facilitated nerve regeneration and improved walking function was also observed when SMDSCs were applied to an acellular conduit to enhance nerve gap bridging in the same study.

Lavasani et al. [53] used human SMDSCs to repair a critical-sized sciatic nerve injury in a mouse model. Transplanted human SMDSCs surrounded the axonal growth cone, while those infiltrating the regenerating nerve differentiated into myelinating Schwann cells. Engraftment of human SMDSCs into the area of the damaged nerve promoted axonal regeneration, which led to functional recovery as measured by sustained gait improvement. Furthermore, no adverse effects were reported in these animals up to 18 months after transplantation. Following human SMDSC therapy, gastrocnemius muscles from mice exhibited substantially less muscle atrophy, an increase in muscle mass after denervation, and reorganization of motor endplates at the postsynaptic sites compared with those from control mice.

### 3.4. Dental Pulp Stem Cells (DPSCs)

The dental pulp contains connective tissue, mesenchymal cells, neural fibres, blood vessels, and lymphatics [54] as well as DPSCs that can self-renew and undergo multidifferentiation [55]. Due to their neural crest origin (in common with cells forming the peripheral nervous system) [56], DPSCs may be more amenable to neural and glial cell differentiation than other stem cell sources [57]. In fact, even in an undifferentiated state, human DPSCs* in vitro* expressed markers such as S100 and nerve growth factor receptor p75 and were able to produce and secrete a range of neurotropic factors including vascular endothelial growth factor (VEGF), brain-derived neurotrophic factor (BDNF), and glial-derived neurotrophic factor (GDNF) [58]. This makes DPSCs an attractive candidate for the treatment of PNI [59].

Sasaki et al. [60] used a silicone tube containing a collagen gel embedded with rat dental pulp cells to repair a gap in the rat facial nerve. Twelve days after transplantation, defective facial nerves connected with silicone tubes containing dental pulp cells were able to support axonal growth to a greater extent than with tubes containing the collagen gel alone. The regenerated nerves contained myelinated fibres and retrograde tracing demonstrated the presence of Fluoro-Gold-positive motor neurons in the facial nucleus of the rat brain. Although this work was undertaken with dental pulp cells as a mixed population, rather than isolated DPSCs, it illustrates the potential of DPSCs in peripheral nerve regeneration.

In a more recent study, Martens and colleagues [58] evaluated the potential of human DPSCs to differentiate towards a Schwann cell lineage. Schwann cell differentiation was examined at the morphological and ultrastructural level and the differentiated human DPSCs showed glial marker expression and secreted neurotrophic factors that promoted sensory neuron survival and neurite outgrowth* in vitro*. In addition, neurites were myelinated by the differentiated human DPSCs in an aligned 3-dimensional coculture system and an engineered neural tissue construct containing aligned human DPSCs in stabilised collagen hydrogels further supported and guided neurite outgrowth.

### 3.5. Hair Follicle Stem Cells (HFSCs)

Stem cells from hair follicles are attractive candidates for use in cell therapy [61], and different populations of HFSCs have been identified [62–64].

Li et al. [62] reported nestin-expressing stem cells in the bulge area of hair follicles which were subsequently shown to be able to differentiate into various nonfollicle cell types and are now known as hair follicle-associated pluripotent stem cells [65]. Mouse green fluorescent protein positive HFSCs were implanted into the gap region of a severed sciatic nerve in a mouse model [66]. The HFSCs differentiated largely into Schwann cells, which produced myelin sheaths around the host axons. Function of the repaired sciatic nerve was measured by contraction of the gastrocnemius muscle upon electrical stimulation. Additionally, HFSCs were implanted in the gap of a severed tibial nerve in a mouse model, resulting in improved walking outcome measures. In a more recent study by Amoh et al. [67], human HFSCs were transplanted around the impinged sciatic nerve in a mouse model, where they differentiated into GFAP-positive cells that associated with host axons. Eight weeks after the transplantation of human HFSCs, gastrocnemius muscle contraction was recorded upon electrical stimulation of the repaired sciatic nerves. Lin et al. [68] differentiated rat HFSCs into Schwann cells* in vitro* using NRG1. These cells, together with neurons, were injected into a decellularised scaffold and cultured* in vitro*. Survival and proliferation of seeded cells as well as neuron-Schwann cell contacts in the scaffolds were observed for at least eight weeks.

Recently, Sakaue and Sieber-Blum isolated human epidermal neural crest stem cells from the hair follicle bulge and showed they can be differentiated into functional Schwann cells [69]. Manipulation of WNT, sonic hedgehog, and tumour-growth-factor *β* signalling pathways and exposing the cells to growth factors led to the expression of Schwann cell markers. Further gene expression profiling indicated the expression of neurotropic and angiogenic transcripts, making these cells promising candidates for nerve repair.

### 3.6. Skin Derived Precursors (SKPs)

Toma et al. [70] reported that multipotent adult stem cells isolated from mammalian dermis can proliferate and differentiate in culture to produce neurons, glia, smooth muscle cells, and adipocytes. SKPs exhibit many similarities to embryonic neural crest stem cells (NCSCs) [71]. McKenzie et al. [72] differentiated SKPs into Schwann cells using medium containing forskolin and NRG1. The Schwann cells were able to proliferate and to express myelin proteins in coculture with sensory neurons. Following transplantation into a mouse sciatic crush model, SKPs associated with and myelinated the host axons within six weeks.

Park et al. [73] investigated the* in vivo* peripheral nerve regeneration potential of autologous porcine SKPs in fibrin glue and collagen tubes following transplantation into a femoral nerve defect miniature pig model. The transplanted cells survived for at least four weeks and histologically complete nerve bundles were observed in the regenerated nerve tissues. Additionally, higher levels of S100 and p75 were detected in the treated animals than in the controls. Khuong et al. [74] studied the effects of SKPs on acute and chronic nerve repair as well as a nerve gap injury repaired with a nerve graft in rats. When used as an adjunct to standard microsurgical nerve repair, SKPs improved outcome in all three scenarios.

A novel case report by Grimoldi et al. [75] used a collagen tube filled with autologous skin derived stem cells to repair the motor and sensory nerves of the left arm of a 23-year-old female patient. Motor and sensory functions of the median nerve demonstrated ongoing recovery after implantation during the three-year follow-up period. Functional recovery of injured median and ulnar nerves was assessed by pinch gauge test and static two-point discrimination and touch test with monofilaments, along with electrophysiological and magnetic resonance imaging (MRI) examinations.

### 3.7. Neural Stem Cells (NSCs)

NSCs have been isolated from both the embryonic and the adult central nervous system [76–78]. Liard et al. [79] transplanted adult pig subventricular zone NSCs inside an autologous venous graft into a femoral nerve gap in an adult pig model and reported improved functional recovery at 6 months compared to controls. Postmortem immunohistochemistry revealed neurosphere-derived cells that survived inside the venous graft from 10 to 240 days and all displayed a neuronal phenotype. Moreover, NSC transplantation increased 2′,3′-cyclic nucleotide 3′-phosphodiesterase (CNPase) expression, indicating activation of intrinsic Schwann cells.

Ni et al. [80] utilised poly(D, L-lactic acid) (PLA) conduits with immobilized fibroblast growth factor 1 (FGF1) and seeded with adult mouse NSCs to bridge a critical size sciatic nerve gap in a rat model. Axon regeneration and functional recovery were observed and evaluated by histology, walking track analysis, and electrophysiology for up to 12 weeks after implantation. In a recent study by Jenkins and colleagues [81], human NSCs were induced from embryonic stem cells and were cultured on an electrospun nerve guidance conduit to evaluate its ability to promote neuronal growth and axonal extension. NSCs survival, migration, and guided neurite extension were observed.

Fu et al. [82] used two recombinant mammalian vectors containing either rat GDNF gene or BDNF gene to transfect adult mouse NSCs. The transfected NSCs showed significantly enhanced expression of GDNF or BDNF mRNA. The transfected NSCs were seeded onto a PLA conduit and implanted into a rat model of sciatic nerve transection. Improved regeneration, myelination, and functional recovery were associated with conduits seeded with the transfected NSCs.

Johnson et al. [83] implanted C17.2 cells, an immortalized mouse NSC line, into three different sciatic nerve injury rat models. Twelve of the forty-five animals used in the study developed large tumours resembling neuroblastomas at the site of the NSC transplants, precluding meaningful interpretation of functional outcome or muscle mass preservation in either the sciatic nerve transection and repair or the crush injury models. The tumour formation was thought to occur as a result of the accumulation of growth factors secreted in high concentrations by the C17.2 cells. Additionally, the transplanted NSCs themselves could have undergone excessive proliferation without the desired differentiation. The authors suggest that further characterization of the interaction of these cells with surrounding tissues of the peripheral nervous system has to be carried out before clinical translation of this approach.

### 3.8. Induced Pluripotent Stem Cells (iPSCs)

Takahashi and Yamanaka [84] used four transcription factors, namely, Oct3/4, Sox2, c-Myc, and Klf4, to generate pluripotent cells, subsequently called iPSCs directly from mouse embryonic or adult fibroblast cultures. Parameters such as factor stoichiometry and culture medium and supplementation have been demonstrated to affect the quality of the iPSCs produced [85].

Wang et al. [45] derived neural crest stem cells from human iPSCs and embryonic stem cells. The neural crest stem cells were seeded into nanofibrous tubular scaffolds (electrospun poly(L-lactide-co-caprolactone)) and used as a bridge for transected sciatic nerves in a rat model. Electrophysiological analysis showed that neural crest stem cell-engrafted nerve conduits resulted in an accelerated regeneration of sciatic nerves at one month when compared with controls. Histological analysis demonstrated that neural crest stem cell transplantation resulted in differentiation into Schwann cells that were able to myelinate the host axons. No teratoma formation was observed for up to one year after neural crest stem cell transplantation* in vivo*. Similar results were obtained by Uemura et al. [86] who examined the long-term outcome of transplanting iPSC-derived neurospheres within nerve conduits for peripheral nerve repair in mice. They confirmed that no teratoma formation was observed up to 48 weeks after transplantation, and axonal regeneration and myelination were enhanced.

Ikeda et al. [87] attempted to repair a sciatic nerve defect in a mouse model by using a bioabsorbable nerve conduit containing both iPSC-derived neurospheres and a basic fibroblast growth factor delivery system. The iPSCs were cultured and differentiated into primary neurospheres containing neural stem cells then secondary neurospheres, which according to Nori et al. [88] differentiated mainly into glial lineage cells. Axon regeneration and functional recovery in the mice were reported to be improved 12 weeks after reconstruction when this combination approach was used.

The derivation of iPSCs from somatic cells provides much potential for patient-specific cell therapies, which bypasses immune rejection issues and ethical concerns associated with using embryonic stem cells as a cell source. However, many important issues need to be addressed in order to use iPSCs in neural tissue engineering, such as differences among iPSC populations in differentiation and expansion and the appropriate differentiation stage of the cells for specific tissue engineering applications [45].

### 3.9. Adipose Derived Stem Cells (ADSCs)

Adipose tissue is largely comprised of adipocytes as well as a smaller stromal vascular fraction which includes ADSCs [89]. These mesenchymal stem cells can be isolated and have been shown to differentiate into cell types of all three germ layers* in vitro* [90, 91]. ADSCs have been extensively studied as an adjunct to nerve repair [92].********

di Summa et al. [93] compared adult rat ADSCs and BMSCs each differentiated to a Schwann cell-like phenotype for the repair of a sciatic nerve injury in a rat model. The mesenchymal stem cells were differentiated into Schwann cell-like cells using a combination of PDGF, BFGF, forskolin, and NRG1. The resulting Schwann cell-like cells from both sources enhanced regeneration but, unlike BMSCs, ADSCs can be harvested less invasively with a higher yield and can be rapidly expanded* in vitro* and show low immunogenicity [92].******

Erba et al. [94] investigated the ability of undifferentiated rat ADSCs in a poly-3-hydroxybutyrate nerve conduit to enhance axonal growth as well as their ability to differentiate* in situ* in a rat sciatic transection model (10 mm gap, 2 weeks). The ADSCs increased regeneration and Schwann cell proliferation compared to controls. However, 14 days after transplantation, a lack of viable implanted cells was observed. As a result, the authors were unable to detect any* in situ* differentiation of ADSCs into neuronal or glial cell types. Similar results were obtained by Santiago et al. [95], who reported that while the transplantation of human ADSCs in a rat sciatic nerve defect promoted nerve tissue regeneration and a decrease in muscle atrophy, the ADSCs did not differentiate to Schwann cell-like cells at the site of injury. This suggests that the regenerative effect of transplanted ADSCs is likely due to an initial boost of released growth factors as well as an indirect effect on endogenous Schwann cell activity. Further evidence for peripheral nerve regeneration through the paracrine effects of ADSCs is presented by Widgerow et al. [96] and Kingham et al. [97].******

Tomita et al. [98] differentiated human ADSCs into Schwann cell-like cells and transplanted them into a rat model of tibial nerve crush, where they closely associated with host axons. The presence of myelin basic protein was detected 8 weeks after transplantation and improved myelin formation was observed with the Schwann cell-like cells compared with undifferentiated ADSCs. * *In a recent study, Georgiou et al. [99] used differentiated rat adipose derived stem cells to construct engineered neural tissue through a combination of cellular self-alignment and plastic compression in a collagen hydrogel. The sheets of aligned cellular collagen supported axon regeneration over a critical length gap (15 mm, 8 weeks) in rat sciatic nerves. Interestingly, the phenotype of the differentiated ADSCs changed when they were transferred to the three-dimensional collagen environment from cell culture flasks, with an apparent increase in expression of key growth factors associated with the support of regeneration.

### 3.10. Perinatal Stem Cells

Perinatal tissues are a potentially useful source of stem cells for tissue engineering purposes as they can be collected in great numbers without causing harm to the donor. Multipotent stem cells have been derived from the placenta, amniotic fluid, amniotic membrane, and umbilical cord [100]. Foetal tissue age rarely exceeds 42 weeks and therefore, in comparison to adult sources, perinatal cells can have less accumulated genetic damage [101].

Matsuse et al. [102] induced human umbilical cord mesenchymal stem cells (UC-MSCs) to differentiate into Schwann cell-like cells then seeded these in transpermeable tubes and transplanted into transected sciatic nerves in adult rats. The Schwann cell-like cells reportedly myelinated the regenerated axons and promoted functional recovery for 21 days after transplantation. Similar results were also obtained by Kuroda et al. [103] and Pereira et al. [104]. In addition to differentiating to a Schwann cell phenotype and expressing Schwann cell markers, Peng and colleagues [105] reported that Schwann cell-like cells derived from human UC-MSCs released BDNF, NGF, and neurotrophin-3* in vitro*. When these cells were cocultured with PC12 cells, neurite outgrowth was observed.

Pan et al. [106] evaluated the effects of neurotrophic factors secreted by rat amniotic fluid mesenchymal stem cells on regeneration of sciatic nerve after crush injury in a rat. Rat amniotic fluid mesenchymal stem cells were embedded in fibrin glue and wrapped around the injured nerve in woven Surgicel®. High levels of expression of BDNF, GDNF, ciliary neurotrophic factor, NGF, and neurotrophin-3 were demonstrated in the amniotic fluid mesenchymal stem cells. Motor function recovery, compound muscle action potential, and nerve conduction latency showed significant improvement in rats treated with amniotic fluid mesenchymal stem cells. Additionally, a high level of expression of S100 and GFAP was observed at the crush site. Similar results were obtained by Li et al. [107] who transplanted amniotic stem cells into a mouse model of a crushed sciatic nerve. Additionally they reported that the stem cells augmented blood perfusion, increased intraneural vascularity, and localised to the perineurium.

## 4. Clinical Translation of Stem Cells: Insights for Peripheral Nerve Repair

### 4.1. Stem Cells Used in Active Clinical Trials

**Figure 1:**
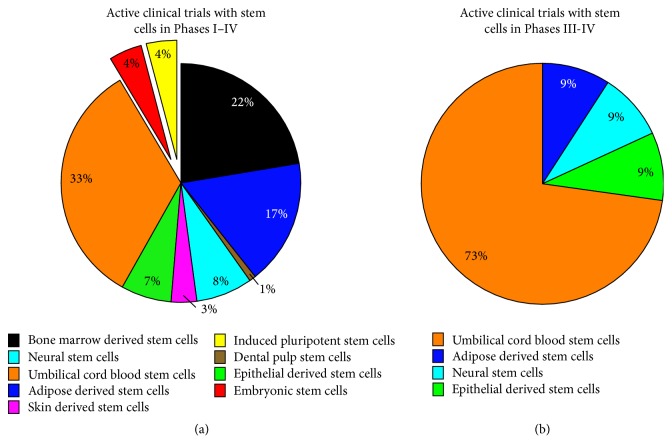
Summary of active (nonrecruiting) clinical trials using stem cell sources internationally. The search was performed on the website of ClinicalTrials.gov (https://clinicaltrials.gov/) on July 28, 2015, and results show there are 117 active studies (a) with 11 studies currently in Phase III or Phase IV clinical trials. Keywords included “Bone marrow derived stem cells” (*n* = 26), “Adipose derived stem cells” (*n* = 20), “Dental pulp stem cells” (*n* = 1), “Neural stem cells” (*n* = 9), “Skin derived stem cells” (*n* = 4), “Epithelial derived stem cells” (*n* = 8), “Umbilical cord blood stem cells” (*n* = 39), “Embryonic stem cells” (*n* = 5), and “Induced pluripotent stem cells” (*n* = 5). With regard to the Phase III and Phase IV trials (b) umbilical cord blood stem cells are the most common stem cells sources (*n* = 8) followed by adipose derived stem cells (*n* = 1), neural stem cells (*n* = 1), and epithelial stem cells (*n* = 1).

As of July 2015, the clinical trials database (https://clinicaltrials.gov/) showed 117 clinical trials using human stem cells internationally and 11 of those trials had reached Phase III and Phase IV status (Figure 1). Of these 11, umbilical cord blood stem cells are the most common source (8 trials) followed by adipose derived stem cells, neural stem cells, and epithelial stem cells (1 trial of each). Cogle et al. [108] and G. D. Fischbach and R. L. Fischbach [109] have reviewed the underlying regulatory, ethical and legal barriers to clinical translation of stem cells and more specifically Walsh and Midha [110] have discussed the practical considerations of using stem cells for peripheral nerve repair.

### 4.2. Regulatory Considerations

Currently, there are no licensed stem cell based artificial nerve tissues for peripheral nerve repair. Using stem cells in clinical trials requires formal approval by the relevant regulatory bodies that are responsible for ensuring that therapies meet standards of safety and quality without jeopardising public health and national security. Berger et al. [111] have recently compared regulatory frameworks with regard to stem cell-based therapies. Their review shows that USA and Europe have the largest number of clinical trials using stem cells followed by East Asia. More notably, since the discovery of induced pluripotent stem cells [84], Japan, which has a similar regulatory framework to USA and Europe, has refined its guidelines to push forward the development of stem cell innovations and has led to the first-ever clinical trial using iPSCs [112].

In Europe, the European Medicines Agency (EMA) regulates stem cell-based therapies and related tissue-engineered medicinal products such as those developed for peripheral nerve repair. Such cellular constructs would be regulated as Advanced Therapy Medicinal Products (ATMPs) [113]. In the UK, the Medicines Health and Regulatory Agency (MHRA) is the competent authority responsible for ATMPs and related clinical trials. For an ATMP to achieve regulatory approval in Europe it would be subject to a centralized marketing procedure whereby applications would be assessed by the Committee for Advanced Therapies and the Committee for Medicinal Products for Human Use before a decision is made by the EMA to grant or refuse marketing authorisation.

In the USA, stem cells and tissue-based devices are regulated by the Food and Drug Administration (FDA) as Human Cells and Tissue-Based Products (HCT/Ps). For peripheral nerve repair application, cells are often combined with natural or synthetic materials to form artificial nerve tissue and are classified as “biological drugs.” These are regulated by the Center for Biologics Evaluation and Research. Whether it be for clinical trials or commercialising a product such as artificial nerve tissue constructs, authorisation must be sought from the FDA through submission of a Biologics License Application. The regulatory framework for Japan is similar to that of Europe and USA [111] and guidelines governing the translation of stem cell therapies were established by the Ministry of Health, Labor and Welfare (MHLW). Kawakami et al. [114] discuss the regulatory impacts of stem cell research in Japan; however, currently a specific framework regulating tissue-engineered products does not exist.

Japan has recently passed a Regenerative Medicine Promotion Law, which provides the opportunity for patients to be treated with regenerative therapies based on the latest scientific knowledge, thus permitting earlier patient access. Further details about the new regulatory system for stem cell therapies in Japan have recently been reviewed by Hara and colleagues [115]. The USA have also introduced schemes to accelerate the delivery of stem cell-based therapies such as “Fast Track,” “Breakthrough Therapy,” “Accelerated Approval,” and “Priority Review” [116] and Knoepfler [117] thoroughly reviews emerging regulatory trends for new stem cell therapies in the USA. Another regulatory pathway for earlier patient access has recently been proposed by Caplan and West [118]. Such schemes to provide earlier patient access are also gaining traction in Europe via the adaptive pathway approach. March of 2014 saw the EMA inviting companies to participate in pilot project adaptive pathways aiming at well-defined patient groups with serious conditions and unmet medical needs [119].

### 4.3. Manufacture and Cell Processing

Obtaining formal approval for a clinical trial from the aforementioned regulatory bodies requires aligning manufacture and cell processing with current Good Manufacturing Practice (GMP), Good Tissue Practice, and Good Clinical Practice. The practice guidelines encompass everything from procurement of stem cells to assessing long-term feasibility and scalability.

A key regulatory issue to consider in the development of stem cell-based therapies for peripheral nerve repair is the term “minimal manipulation.” This is defined as “processing that does not alter the biology of the cell” [120]; however, stem cell therapies often require multistage processing especially for nonhomologous use. From a tissue engineering perspective, the stem cells would often be differentiated* in vitro* prior to being combined with a scaffolding material, for example, collagen to form artificial tissue [99, 121], and therefore the tissue-engineered device would be classed as more than minimally manipulated. Unger et al. [122] reviewed the pivotal role of GMP with regard to hESCs lines; however, there are still challenges to develop a standard operating procedure for their derivation. Reviews by Bieback et al. [123] and more recently Sharma et al. review clinical scale manufacturing and translation of mesenchymal stromal cells with a focus on regulatory frameworks and GMP.

Several authors also comment on the lack of regulatory harmonisation where subtle differences in GMP protocols can prevent a certain therapy moving forward [124–126]. For example, these differences have been noticed in sterilising procedures, control of starting materials, and disparities in definitions of quality, safety, and efficacy. The International Society for Stem Cell Research (ISSCR) set out guidelines describing how stem cell therapies can be responsibly translated [127]. Abiding by these international guidelines also offers a certain degree of standardisation since all stem cells will be manufactured in a reproducible manner to meet present specifications that ensure efficacy and safety.

### 4.4. Stem Cell Extraction and Isolation

Stem cells can be obtained from various sources, with each technique presenting different challenges. In light of this, the European commission has issued directives to ensure cross-border harmonisaton in stem cell donation, procurement, processing and preservation [128]. Here, some of the differences in obtaining stem cells from various sources will be briefly discussed.

In the UK, the Human Fertilisation and Embryology Authority (HFEA) is an independent regulator responsible for the procurement of gametes and the associated processing involved in the creation of an embryo and this includes overseeing the derivation of embryonic stem cell lines [129]. Once the inner cell mass is disassociated from the embryo, the originator is obliged to donate samples to the United Kingdom Stem Cell Bank (UKSCB) and the stem cell line is regulated by the Human Tissue Authority [130]. Isolation of mesenchymal stem cells can involve multistage* ex vivo* processing and numerous protocols are available [131–135].

Umbilical cord blood is commonly collected by inserting a cannula into an umbilical vein and allowing the blood to drain out; this is often known as gravity-assisted collection [136]. More recent attention is beginning to focus on other neonatal tissues that can be clinically translated, for example, Wharton's jelly of the umbilical cord, amniotic membrane, and placenta [137–139].

The most widely used method of extracting bone marrow is through a bone marrow biopsy commonly from the posterior superior iliac spine or crest usually under general anesthetic [140, 141]. The BMSCs reside in the bone marrow stroma in small quantities [142] and this number decreases with age; hence, using this as an autologous source of stem cells to treat an elderly population would be challenging [143]. The abundance and accessibility of adipose tissue make the ADSCs a potentially more attractive mesenchymal stem cell source [144]. Large quantities of subcutaneous adipose tissue can be obtained through minimally invasive liposuction surgery usually under local anesthetic with minimal donor site morbidity.

Dental pulp stem cells can be isolated from discarded wisdom or deciduous teeth therefore bypassing the need for invasive tissue harvest associated with other stem cell sources [145]. Teeth contain various different populations of stem cells that can be isolated in different ways and stored in stem cell banks. Martens and colleagues [146] have reviewed dental stem cells and their potential role in neural regeneration.

While autologous adult neural stem cells can be harvested and may be useful for peripheral nerve regeneration, their extraction requires intricate surgery from either the dentate gyrus of the hippocampus or the subventricular zone of the lateral walls of the lateral ventricles of the brain [147]. The invasiveness of the procedure together with the fact that they are present in low numbers may limit their clinical applications [148]. Compared to adult NSCs, embryonic and foetal NSCs are relatively easily cultured and grown [149], and the majority of NSC lines available today are of foetal origin [150].

Other stem cell sources being investigated for use in peripheral nerve tissue engineering include the hair follicle stem cells and skin derived stem cells [67, 71]. The use of hair follicle stem cells in particular for nerve regeneration can potentially overcome problems associated with other stem cell sources as they can be easily harvested and are not associated with so many ethical issues [151].

### 4.5. Stem Cell Expansion

The addition of additives such as growth factors can differentiate a stem cell towards a particular lineage [152] although the large-scale amplification of stem cells also increases the mutation rate that could directly impact treatment with these cells [152, 153]. Recent work has also shown that long-term culture can alter the genetic composition of the cells [154, 155].

Currently, there is no optimal basal medium used for stem cell expansion. Some studies use Iscove's Modified Dulbecco's Media [36, 38], whereas others use Dulbeccos Modified Eagle's Media [52, 156] or alpha-minimum essential medium [157]. Serum appears to be a key component for the expansion of some cells; hence, many media are supplemented with GMP grade Foetal Bovine Serum (FBS) to produce a formulated cocktail of proteins, growth factors, and nutrients. FBS is widely used throughout research and clinical studies although its commercial use is undesirable due to several inherent problems outlined in [158]. These include, but are not limited to, cross-species contamination, high content of xenogeneic proteins, and high batch-to-batch variation which all increase the regulatory burden. Nevertheless, the FDA has approved the use of GMP clinical grade FBS; however, [159, 160] show that immunogenicity against FBS proteins reduces the therapeutic benefit. With a focus on clinical translation, recent approaches have begun to explore defined serum-free and nonxenogeneic media options for multiple cell lineages [161–163]. Various bioprocessing approaches have been developed to support the large-scale expansion of therapeutic cells, including multilayer cell factories and closed-system automated bioreactors in accordance with GMP guidelines; these are detailed in [164].

### 4.6. Variability in Stem Cells

Recent literature describes the importance of donor age and time in culture in determining mesenchymal stem cell efficacy and quantity [165–167]. For example, a study by Siegel et al. [168] isolated BMSCs from 53 donors, aged between 13 and 80 years, and found that age and gender affect expression of certain makers and consequently BMSC function. A study by Choudhery et al. [169] recently described the negative effect of age on ADSCs whereby aged MSCs displayed senescent features, reduced viability, and proliferation and differentiation potential when compared to ADSCs from young donors. Conversely, a study by Choi et al. [170] used tonsil-derived mesenchymal stem cells and suggested that donor age and long-term passage had little effect.

Emerging studies have begun to understand the limitations of* in vitro* stem cell assays since it is becoming increasingly apparent that* in vitro* data do not correlate with* in vivo* results [171, 172].

There are also differences in the proliferation capacity of stem cells; for example, to generate a culture that is confluent in approximately 5–7 days, it is common to seed freshly isolated bone marrow stem cells in the range of 20,000–400,000 cells/cm^2^ whereas ADSCs only require to be seeded at a density of 3500 cells/cm^2^ to achieve the same level of confluence in the same time [144]. Considering this, Fossett and Khan [173] have recently discussed methods to optimise hMSC numbers for clinical application. A short communication by Zhou et al. [174] describes that seeding density of mouse embryonic stem cells and also quality of embryonic bodies directly effect the neural differentiation of the cells.

### 4.7. Preservation

Following isolation of the desired stem cells they can be either directly used for therapy or stored frozen. The cryopreservation of stem cells including induced pluripotent stem cells with consideration of GMP is reviewed by Hunt [175]. Currently, the gold standard method for cryopreservation involves suspending embryonic or mesenchymal stem cells in a mixture of FBS, growth media, and usually 10% dimethylsulfoxide (DMSO). This solution undergoes a slow-freeze protocol; however, thawing is rapid. Interestingly, this protocol is effective for murine and embryonic stem cells; however, storing human embryonic stem cells under these conditions is plagued with poor cell survival [176]. Various cryopreservation protocols for embryonic stem cells have been reviewed by [177, 178]. Different research groups are optimising preservation protocols with novel techniques to ensure maximal cell viability after thaw [179, 180]. Similarly, numerous studies investigated the effects of cryopreservation conditions on MSCs and demonstrated that they can be stored without loss of function [181]. In fact, a study by Ginis et al. [182] showed that BMSCs can be successfully preserved for the long term and delivered to the clinic in protective hypothermic storage in the short term. There are concerns about the toxicity of DMSO in humans; thus, there is substantial interest in reducing or completely removing DMSO from the preservation solution [183, 184].

## 5. Conclusion

PNI continues to be an area of unmet clinical need and advances in tissue engineering would be a welcome therapeutic option. To this end, a number of studies have highlighted the ability of stem cells from a variety of sources to differentiate into Schwann cells as starting material for these constructs. This competitive environment is both healthy and beneficial; each different type of stem cell will have its own unique set of advantages and disadvantages and success may ultimately lie in selecting the correct source for the desired tissue engineering strategy. Although there is currently no licensed stem cell based product for PNI, it is important that we take these issues into account at an early stage in development. It is highly encouraging that there is a clear international effort to develop and characterize stem cells for Schwann cell based engineered tissue for therapeutic application within the PNI field.

## Figures and Tables

**Table 1:** Summary of current evidence assessing the effect of different stem cell types on peripheral nerve regeneration in animal models.

Stem cell source tissue	Author	Experimental model	Mode of delivery	Therapeutic cell properties	Key outcome measures
Adipose	Georgiou et al. [99]	Rat sciatic transection (15 mm gap, 8 weeks)	Aligned cellular collagen constructs in a collagen tube	Differentiated rat ADSCs	Axon regeneration, myelination.
	Hsueh et al. [185]	Rat sciatic transection (10 mm gap, 6 weeks)	Neurospheres seeded in a chitosan-coated silicon conduit	Xenogeneic hADSC neurospheres	Axon regeneration, myelination, inflammation, intraneural scarring, muscle fibres, and gait.
	Kingham et al. [97]	Rat sciatic transection (10 mm gap, 2 weeks)	Cells in fibrin matrix seeded in a fibrin conduit	Stimulated xenogeneic hADSCs	Axon regeneration, vascularisation, cell survival, and gene expression changes in DRG and spinal cord.
	Scholz et al. [186]	Athymic rat sciatic transection (13 mm gap, up to 4 months)	Cells in culture medium seeded in a silastic conduit	Xenogeneic differentiated hADSCs	Axon regeneration, extensor postural thrust, sensory evaluation, and muscle weight.
	Carriel et al. [187]	Rat sciatic transection (10 mm gap, 12 weeks)	Cells in a fibrin-agarose hydrogel seeded in a collagen conduit	Autologous ADSCs	Axon regeneration, myelination, electrophysiology, pinch test, and toe spread.
	Mohammadi et al. [33]	Rat sciatic transection (10 mm gap, up to 12 weeks)	Cells in PBS seeded in a silicone conduit	Allogeneic ADSCs	Nerve fibres, walking track analysis, and muscle weight.
	Suganuma et al. [188]	Rat sciatic transection (10 mm gap, 2 weeks)	Cells in type I collagen gel seeded in a silicone conduit	Autologous uncultured ADSCs	Axon regeneration, Schwann cell infiltration, cell survival, and gene expression in repaired tissue.
	Orbay et al. [189]	Rat sciatic transection (10 mm gap, up to 6 months)	Cells in collagen gel seeded in a silicone conduit	Autologous ADSCs differentiated into Schwann cell-like cells	Nerve fibres, vascularisation, cell survival, walking track analysis electrophysiology, and muscle weight.
	Liu et al. [190]	Rat sciatic transection (15 mm gap, 12 weeks)	Cells in culture medium seeded in an acellular nerve allograft	Allogeneic undifferentiated ADSCs	Footprint analysis, sciatic functional index, electrophysiology, myelination, and nerve fibre density.
	Reid et al. [191]	Rat sciatic transection (10 mm gap, 2 weeks)	Cells in culture medium seeded in a PCL conduit	Allogeneic ADSCs differentiated into Schwann cell-like cells	Gene expression changes in the DRG.
	Shen et al. [192]	Rat sciatic transection (10 mm gap, up to 8 weeks)	Cells in culture medium seeded in a GGT conduit	Allogeneic undifferentiated ADSCs	Electrophysiology, walking track analysis, footprint analysis, nerve fibres, and myelination.
	Sun et al. [193]	Rat facial transection (8 mm gap, 8 weeks)	Cells in culture medium seeded in a decellularised allogeneic artery conduit	Autologous ADSCs differentiated into Schwann cell-like cells	Functional evaluation of vibrissae movement, electrophysiology, morphological evaluation of regenerated nerve segments, and retrograde labelling of facial motor neurons.
	Wei et al. [194]	Rat sciatic transection (10 mm gap, up to 24 weeks)	Cells in culture medium seeded onto a chitosan/silk fibroin conduit	Allogeneic undifferentiated ADSCs	Walking track analysis, footprint analysis, muscle mass, axon regeneration, and myelination.
	di Summa et al. [93]	Rat sciatic transection (10 mm gap, 2 weeks)	Cells in culture medium seeded in a fibrin conduit	Allogeneic ADSCs differentiated into Schwann cell-like cells	Axon regeneration, Schwann cell infiltration.
	Erba et al. [94]	Rat sciatic transection (10 mm gap, 2 weeks)	Cells in fibrinogen seeded in a PHB conduit	Allogeneic undifferentiated ADSCs	Axonal regeneration, transplanted cell tracking.
	Zhang et al. [195]	Rat sciatic transection (10 mm gap, 3 months)	Cells in culture medium seeded in a xenogeneic acellular graft	Autologous ADSCs differentiated to a neuronal phenotype	Axon regeneration, myelination, and electrophysiology.
	Ghoreishian et al. [196]	Dog facial nerve transection (7 mm gap, 12 weeks)	Cells in an alginate hydrogel seeded in a GORE-TEX® conduit	Autologous undifferentiated ADSCs	Axon regeneration, electrophysiology.

Bone marrow	Ding et al. [36]	Dog sciatic transection (50 mm gap, 6 months)	Cells in culture medium seeded in a chitosan/PLGA conduit	Autologous undifferentiated BMSCs	Electrophysiology, muscle mass, axon regeneration, myelination, and vascularisation.
	Xue et al. [37]	Dog sciatic transection (60 mm gap, 12 months)	Cells in saline seeded in a chitosan/PLGA conduit	Autologous undifferentiated BMSCs	Posture and gait analysis, electrophysiology, muscle mass, myelination, and nerve fibres.
	Sakar et al. [197]	Rat sciatic transection (10 mm gap, up to 8 weeks)	Cells in PBS seeded in a PHBHHx nerve graft	Allogeneic undifferentiated BMSCs	Electrophysiology, axon regeneration, myelination, walking track analysis, and vascularisation.
	Hu et al. [38]	Monkey median transection (50 mm gap, 1 year)	Cells in culture medium seeded in a chitosan/PLGA conduit	Autologous undifferentiated BMSCs	Behaviour observation, electrophysiology, myelination, safety evaluation, and axon regeneration.
	Wakao et al. [39]	Monkey median transection (20 mm gap, 1 year)	Cells in a collagen sponge seeded in a biodegradable conduit	Autologous BMSCs differentiated to Schwann cell-like cells	Cell proliferation for local tumour formation, electrophysiology, hand movement analysis, immunocytochemistry, general health follow-up, immune response, myelination, and axon regeneration.
	Oliveira et al. [156]	Mouse median transection (2 mm gap, 8 weeks)	Cells in culture medium seeded in a PCL conduit	Autologous undifferentiated BMSCs	Myelination, nerve fibres, electrophysiology, muscle mass, creatine phosphokinase levels, grasping test, and immunohistochemistry.
	Ladak et al. [34]	Rat sciatic transection (12 mm gap, 12 weeks)	Cells in culture medium seeded in a biodegradable collagen nerve guide	Autologous BMSCs differentiated to Schwann cell-like cells	Electrophysiology, axon regeneration, and muscle mass.
	Mohammadi et al. [198]	Rat sciatic transection (10 mm gap, up to 12 weeks)	Cells in PBS seeded in an inside-out vein graft	Allogeneic undifferentiated BMSCs	Walking track analysis, immunohistochemistry, muscle mass, axon regeneration, and myelination.

Embryonic	Cui et al. [28]	Rat sciatic transection (10 mm gap, up to 3 months)	Cells in culture medium seeded into the gap between the nerve stumps with the surrounding epineurium as a natural conduit	Xenogeneic mouse ESCs differentiated into neural progenitor cells	Electrophysiology, axon regeneration, and myelination.

Umbilical cord (Wharton's Jelly)	Matsuse et al. [102]	Rat sciatic transection (8 mm gap, up to 21 days)	Cells in Matrigel seeded in transpermeable tubes	Allogeneic umbilical cord stem cells differentiated into Schwann cell-like cells	Walking track analysis, immunohistochemistry, myelination, and axonal regeneration.

Dental pulp	Matsushita et al. [199]	Rat sciatic transection (3 mm gap, up to 32 weeks)	Cells in PBS seeded in a chitosan conduit	Xenogeneic human whole dental pulp	Immunohistochemistry, myelination, and axon regeneration.

Neural tissue	Ni et al. [80]	Rat sciatic transection (15 mm gap, up to 12 weeks)	Cells in culture medium seeded in a PLA conduit	Xenogeneic mouse undifferentiated NSCs	Walking track analysis, sciatic functional index, electrophysiology, revascularisation, axon regeneration, and myelination.
	Liard et al. [79]	Pig femoral transection (30 mm gap, up to 240 days)	Neurospheres seeded in an autologous venous bridge	Allogeneic undifferentiated NSCs	Electromyography, thigh flexion, and phenotypic characterization of grafted cell progeny.

Skin	Park et al. [73]	Pig femoral transection (10 mm gap, up to 4 weeks)	Cells in fibrin glue seeded in a Lyoplant® conduit	Autologous undifferentiated SKPs	Axon regeneration, nerve fibres.

iPSCs	Uemura et al. [86]	Mouse sciatic transection (5 mm gap, up to 48 weeks)	Neurospheres seeded in a PCL/PLA conduit	Mouse iPSCs differentiated to neurospheres	Print length factor, foot withdrawal, histomorphometry, and myelination.
	Wang et al. [45]	Rat sciatic transection (10 mm gap, 1 month)	Cells in Matrigel seeded in a PCL/PLA/sodium acetate conduit	Human iPSCs differentiated to neural crest stem cells	Myelination, axon regeneration, electrophysiology, and cell survival.

ADSCs: adipose derived stem cells; hADSCs: human adipose derived stem cells; DRG: dorsal root ganglion; PBS: phosphate buffered saline; PCL: poly-*ε*-caprolactone; GGT: genipin cross-linked gelatin annexed with tricalcium phosphate ceramic particles; PHB: poly-3-hydroxybutyrate; PLGA: poly(lactic-co -glycolic acid); BMSCs: bone marrow stem cells; PHBHHx: poly(3-hydroxybutyrate-co-3- hydroxyhexanoate); ESCs: embryonic stem cells; PLA: polylactic acid; NSCs: neural stem cells; SKPs: skin derived precursors; iPSCs: induced pluripotent stem cells.

## References

[1] Callaghan B. C., Cheng H. T., Stables C. L., Smith A. L., Feldman E. L. (2012). Diabetic neuropathy: clinical manifestations and current treatments. *The Lancet Neurology*.

[2] van den Berg B., Walgaard C., Drenthen J., Fokke C., Jacobs B. C., van Doorn P. A. (2014). Guillain-Barré syndrome: pathogenesis, diagnosis, treatment and prognosis. *Nature Reviews Neurology*.

[3] Antoine J.-C., Camdessanché J.-P. (2007). Peripheral nervous system involvement in patients with cancer. *The Lancet Neurology*.

[4] Kömürcü F., Zwolak P., Benditte-Klepetko H., Deutinger M. (2005). Management strategies for peripheral iatrogenic nerve lesions. *Annals of Plastic Surgery*.

[5] Saadat S., Eslami V., Rahimi-Movaghar V. (2011). The incidence of peripheral nerve injury in trauma patients in Iran. *Turkish Journal of Trauma & Emergency Surgery*.

[6] Isaacs J., Browne T. (2014). Overcoming short gaps in peripheral nerve repair: conduits and human acellular nerve allograft. *Hand*.

[7] Kingham P. J., Terenghi G. (2006). Bioengineered nerve regeneration and muscle reinnervation. *Journal of Anatomy*.

[8] Chen Z.-L., Yu W.-M., Strickland S. (2007). Peripheral regeneration. *Annual Review of Neuroscience*.

[9] Scheib J., Höke A. (2013). Advances in peripheral nerve regeneration. *Nature Reviews Neurology*.

[10] Hall S. M. (1999). The biology of chronically denervated Schwann cells. *Annals of the New York Academy of Sciences*.

[11] Jessen K. R., Mirsky R., Lloyd A. C. (2015). Schwann cells: development and role in nerve repair. *Cold Spring Harbor Perspectives in Biology*.

[12] Nectow A. R., Marra K. G., Kaplan D. L. (2012). Biomaterials for the development of peripheral nerve guidance conduits. *Tissue Engineering Part B: Reviews*.

[13] Bell J. H. A., Haycock J. W. (2012). Next generation nerve guides: materials, fabrication, growth factors, and cell delivery. *Tissue Engineering Part B: Reviews*.

[14] Wang S., Cai L. (2010). Polymers for fabricating nerve conduits. *International Journal of Polymer Science*.

[15] Gu X., Ding F., Williams D. F. (2014). Neural tissue engineering options for peripheral nerve regeneration. *Biomaterials*.

[16] Herberts C. A., Kwa M. S. G., Hermsen H. P. H. (2011). Risk factors in the development of stem cell therapy. *Journal of Translational Medicine*.

[17] Hyun I. (2010). The bioethics of stem cell research and therapy. *The Journal of Clinical Investigation*.

[18] Thomson J. A., Itskovitz-Eldor J., Shapiro S. S. (1998). Embryonic stem cell lines derived from human blastocysts. *Science*.

[19] Tewarie R. S. N., Hurtado A., Bartels R. H., Grotenhuis A., Oudega M. (2009). Stem cell-based therapies for spinal cord injury. *Journal of Spinal Cord Medicine*.

[20] Vawda R., Wilcox J., Fehlings M. G. (2012). Current stem cell treatments for spinal cord injury. *Indian Journal of Orthopaedics*.

[21] Kim B. G., Hwang D. H., Lee S. I. (2007). Stem cell-based cell therapy for spinal cord injury. *Cell Transplantation*.

[22] Reubinoff B. E., Itsykson P., Turetsky T. (2001). Neural progenitors from human embryonic stem cells. *Nature Biotechnology*.

[23] Ziegler L., Grigoryan S., Yang I. H., Thakor N. V., Goldstein R. S. (2011). Efficient generation of schwann cells from human embryonic stem cell-derived neurospheres. *Stem Cell Reviews and Reports*.

[24] Brokhman I., Gamarnik-Ziegler L., Pomp O., Aharonowiz M., Reubinoff B. E., Goldstein R. S. (2008). Peripheral sensory neurons differentiate from neural precursors derived from human embryonic stem cells. *Differentiation*.

[25] Pomp O., Brokhman I., Ben-Dor I., Reubinoff B., Goldstein R. S. (2005). Generation of peripheral sensory and sympathetic neurons and neural crest cells from human embryonic stem cells. *Stem Cells*.

[26] Lee G., Kim H., Elkabetz Y. (2007). Isolation and directed differentiation of neural crest stem cells derived from human embryonic stem cells. *Nature Biotechnology*.

[27] Jiang X., Gwye Y., McKeown S. J., Bronner-Fraser M., Lutzko C., Lawlor E. R. (2009). Isolation and characterization of neural crest stem cells derived from in vitro-differentiated human embryonic stem cells. *Stem Cells and Development*.

[28] Cui L., Jiang J., Wei L. (2008). Transplantation of embryonic stem cells improves nerve repair and functional recovery after severe sciatic nerve axotomy in rats. *Stem Cells*.

[29] Kim J., Orkin S. H. (2011). Embryonic stem cell-specific signatures in cancer: Insights into genomic regulatory networks and implications for medicine. *Genome Medicine*.

[30] Mathieu J., Zhang Z., Zhou W. (2011). HIF induces human embryonic stem cell markers in cancer cells. *Cancer Research*.

[31] Nussbaum J., Minami E., Laflamme M. A. (2007). Transplantation of undifferentiated murine embryonic stem cells in the heart: teratoma formation and immune response. *The FASEB Journal*.

[32] Lin W., Chen X., Wang X., Liu J., Gu X. (2008). Adult rat bone marrow stromal cells differentiate into schwann cell-like cells in vitro. *In Vitro Cellular and Developmental Biology—Animal*.

[33] Mohammadi R., Azizi S., Amini K. (2013). Effects of undifferentiated cultured omental adipose-derived stem cells on peripheral nerve regeneration. *Journal of Surgical Research*.

[34] Ladak A., Olson J., Tredget E. E., Gordon T. (2011). Differentiation of mesenchymal stem cells to support peripheral nerve regeneration in a rat model. *Experimental Neurology*.

[35] Shen J., Duan X.-H., Cheng L.-N. (2010). In vivo MR imaging tracking of transplanted mesenchymal stem cells in a rabbit model of acute peripheral nerve traction injury. *Journal of Magnetic Resonance Imaging*.

[36] Ding F., Wu J., Yang Y. (2010). Use of tissue-engineered nerve grafts consisting of a chitosan/poly(lactic-co-glycolic acid)-based scaffold included with bone marrow mesenchymal cells for bridging 50-mm dog sciatic nerve gaps. *Tissue Engineering Part A*.

[37] Xue C., Hu N., Gu Y. (2012). Joint use of a chitosan/PLGA scaffold and MSCs to bridge an extra large gap in dog sciatic nerve. *Neurorehabilitation and Neural Repair*.

[38] Hu N., Wu H., Xue C. (2013). Long-term outcome of the repair of 50 mm long median nerve defects in rhesus monkeys with marrow mesenchymal stem cells-containing, chitosan-based tissue engineered nerve grafts. *Biomaterials*.

[39] Wakao S., Hayashi T., Kitada M. (2010). Long-term observation of auto-cell transplantation in non-human primate reveals safety and efficiency of bone marrow stromal cell-derived schwann cells in peripheral nerve regeneration. *Experimental Neurology*.

[40] Wang J., Ding F., Gu Y., Liu J., Gu X. (2009). Bone marrow mesenchymal stem cells promote cell proliferation and neurotrophic function of Schwann cells in vitro and in vivo. *Brain Research*.

[41] Caddick J., Kingham P. J., Gardiner N. J., Wiberg M., Terenghi G. (2006). Phenotypic and functional characteristics of mesenchymal stem cells differentiated along a Schwann cell lineage. *Glia*.

[42] Ao Q., Fung C.-K., Yat-Ping Tsui A. (2011). The regeneration of transected sciatic nerves of adult rats using chitosan nerve conduits seeded with bone marrow stromal cell-derived schwann cells. *Biomaterials*.

[43] Zarbakhsh S., Bahktiari M., Faghihi A. (2012). The effects of Schwann and bone marrow stromal stem cells on sciatic nerve injury in rat: a comparison of functional recovery. *Cell Journal*.

[44] Keilhoff G., Stang F., Goihl A., Wolf G., Fansa H. (2006). Transdifferentiated mesenchymal stem cells as alternative therapy in supporting nerve regeneration and myelination. *Cellular and Molecular Neurobiology*.

[45] Wang A., Tang Z., Park I.-H. (2011). Induced pluripotent stem cells for neural tissue engineering. *Biomaterials*.

[46] Jia H., Wang Y., Tong X.-J. (2012). Sciatic nerve repair by acellular nerve xenografts implanted with BMSCs in rats xenograft combined with BMSCs. *Synapse*.

[47] Raheja A., Suri V., Suri A. (2012). Dose-dependent facilitation of peripheral nerve regeneration by bone marrow-derived mononuclear cells: a randomized controlled study. Laboratory investigation. *Journal of Neurosurgery*.

[48] Tamaki T., Akatsuka A., Ando K. (2002). Identification of myogenic-endothelial progenitor cells in the interstitial spaces of skeletal muscle. *Journal of Cell Biology*.

[49] Tamaki T., Okada Y., Uchiyama Y. (2007). Clonal multipotency of skeletal muscle-derived stem cells between mesodermal and ectodermal lineage. *Stem Cells*.

[50] Deasy B. M., Gharaibeh B. M., Pollett J. B. (2005). Long-term self-renewal of postnatal muscle-derived stem cells. *Molecular Biology of the Cell*.

[51] Urish K. L., Vella J. B., Okada M. (2009). Antioxidant levels represent a major determinant in the regenerative capacity of muscle stem cells. *Molecular Biology of the Cell*.

[52] Tamaki T., Hirata M., Soeda S. (2014). Preferential and comprehensive reconstitution of severely damaged sciatic nerve using murine skeletal muscle-derived multipotent stem cells. *PLoS ONE*.

[53] Lavasani M., Thompson S. D., Pollett J. B. (2014). Human muscle-derived stem/progenitor cells promote functional murine peripheral nerve regeneration. *The Journal of Clinical Investigation*.

[54] Liu H., Gronthos S., Shi S. (2006). Dental pulp stem cells. *Methods in Enzymology*.

[55] Gronthos S., Mankani M., Brahim J., Robey P. G., Shi S. (2000). Postnatal human dental pulp stem cells (DPSCs) in vitro and in vivo. *Proceedings of the National Academy of Sciences of the United States of America*.

[56] Arthur A., Rychkov G., Shi S., Koblar S. A., Gronthose S. (2008). Adult human dental pulp stem cells differentiate toward functionally active neurons under appropriate environmental cues. *Stem Cells*.

[57] Ibarretxe G., Crende O., Aurrekoetxea M., García-Murga V., Etxaniz J., Unda F. (2012). Neural crest stem cells from dental tissues: a new hope for dental and neural regeneration. *Stem Cells International*.

[58] Martens W., Sanen K., Georgiou M. (2014). Human dental pulp stem cells can differentiate into Schwann cells and promote and guide neurite outgrowth in an aligned tissue-engineered collagen construct in vitro. *The FASEB Journal*.

[59] Neirinckx V., Coste C., Rogister B., Wislet-Gendebien S. (2013). Concise review: adult mesenchymal stem cells, adult neural crest stem cells, and therapy of neurological pathologies: a state of play. *Stem Cells Translational Medicine*.

[60] Sasaki R., Aoki S., Yamato M. (2008). Tubulation with dental pulp cells promotes facial nerve regeneration in rats. *Tissue Engineering Part A*.

[61] Sieber-Blum M. (2014). Human epidermal neural crest stem cells as candidates for cell-based therapies, disease modeling, and drug discovery. *Birth Defects Research Part C—Embryo Today: Reviews*.

[62] Li L., Mignone J., Yang M. (2003). Nestin expression in hair follicle sheath progenitor cells. *Proceedings of the National Academy of Sciences of the United States of America*.

[63] Sieber-Blum M., Grim M. (2004). The adult hair follicle: cradle for pluripotent neural crest stem cells. *Birth Defects Research Part C—Embryo Today: Reviews*.

[64] Sieber-Blum M., Grim M., Hu Y. F., Szeder V. (2004). Pluripotent neural crest stem cells in the adult hair follicle. *Developmental Dynamics*.

[65] Yashiro M., Mii S., Aki R. (2015). From hair to heart: nestin-expressing hair-follicle-associated pluripotent (HAP) stem cells differentiate to beating cardiac muscle cells. *Cell Cycle*.

[66] Amoh Y., Li L., Campillo R. (2005). Implanted hair follicle stem cells form Schwann cells that support repair of severed peripheral nerves. *Proceedings of the National Academy of Sciences of the United States of America*.

[67] Amoh Y., Aki R., Hamada Y. (2012). Nestin-positive hair follicle pluripotent stem cells can promote regeneration of impinged peripheral nerve injury. *Journal of Dermatology*.

[68] Lin H., Liu F., Zhang C. (2011). Characterization of nerve conduits seeded with neurons and schwann cells derived from hair follicle neural crest stem cells. *Tissue Engineering Part A*.

[69] Sakaue M., Sieber-Blum M. (2015). Human epidermal neural crest stem cells as a source of schwann cells. *Development*.

[70] Toma J. G., Akhavan M., Fernandes K. J. L. (2001). Isolation of multipotent adult stem cells from the dermis of mammalian skin. *Nature Cell Biology*.

[71] Biernaskie J., Miller F. D. (2010). White matter repair: skin-derived precursors as a source of myelinating cells. *The Canadian Journal of Neurological Sciences*.

[72] McKenzie I. A., Biernaskie J., Toma J. G., Midha R., Miller F. D. (2006). Skin-derived precursors generate myelinating Schwann cells for the injured and dysmyelinated nervous system. *The Journal of Neuroscience*.

[73] Park B.-W., Kang D.-H., Kang E.-J. (2012). Peripheral nerve regeneration using autologous porcine skin-derived mesenchymal stem cells. *Journal of Tissue Engineering and Regenerative Medicine*.

[74] Khuong H. T., Kumar R., Senjaya F. (2014). Skin derived precursor Schwann cells improve behavioral recovery for acute and delayed nerve repair. *Experimental Neurology*.

[75] Grimoldi N., Colleoni F., Tiberio F. (2015). Stem cell salvage of injured peripheral nerve. *Cell Transplantation*.

[76] Fuentealba L. C., Obernier K., Alvarez-Buylla A. (2012). Adult neural stem cells bridge their niche. *Cell Stem Cell*.

[77] Ernst A., Alkass K., Bernard S. (2014). Neurogenesis in the striatum of the adult human brain. *Cell*.

[78] Reynolds B. A., Weiss S. (1992). Generation of neurons and astrocytes from isolated cells of the adult mammalian central nervous system. *Science*.

[79] Liard O., Segura S., Sagui E. (2012). Adult-brain-derived neural stem cells grafting into a vein bridge increases postlesional recovery and regeneration in a peripheral nerve of adult pig. *Stem Cells International*.

[80] Ni H.-C., Tseng T.-C., Chen J.-R., Hsu S.-H., Chiu I.-M. (2013). Fabrication of bioactive conduits containing the fibroblast growth factor 1 and neural stem cells for peripheral nerve regeneration across a 15–mm critical gap. *Biofabrication*.

[81] Jenkins P. M., Laughter M. R., Lee D. J., Lee Y. M., Freed C. R., Park D. (2015). A nerve guidance conduit with topographical and biochemical cues: potential application using human neural stem cells. *Nanoscale Research Letters*.

[82] Fu K.-Y., Dai L.-G., Chiu I.-M., Chen J.-R., Hsu S.-H. (2011). Sciatic nerve regeneration by microporous nerve conduits seeded with glial cell line-derived neurotrophic factor or brain-derived neurotrophic factor gene transfected neural stem cells. *Artificial Organs*.

[83] Johnson T. S., O'Neill A. C., Motarjem P. M., Nazzal J., Randolph M., Winograd J. M. (2008). Tumor formation following murine neural precursor cell transplantation in a rat peripheral nerve injury model. *Journal of Reconstructive Microsurgery*.

[84] Takahashi K., Yamanaka S. (2006). Induction of pluripotent stem cells from mouse embryonic and adult fibroblast cultures by defined factors. *Cell*.

[85] Buganim Y., Markoulaki S., Van Wietmarschen N. (2014). The developmental potential of iPSCs is greatly influenced by reprogramming factor selection. *Cell Stem Cell*.

[86] Uemura T., Ikeda M., Takamatsu K., Yokoi T., Okada M., Nakamura H. (2014). Long-term efficacy and safety outcomes of transplantation of induced pluripotent stem cell-derived neurospheres with bioabsorbable nerve conduits for peripheral nerve regeneration in mice. *Cells Tissues Organs*.

[87] Ikeda M., Uemura T., Takamatsu K. (2014). Acceleration of peripheral nerve regeneration using nerve conduits in combination with induced pluripotent stem cell technology and a basic fibroblast growth factor drug delivery system. *Journal of Biomedical Materials Research Part A*.

[88] Nori S., Okada Y., Yasuda A. (2011). Grafted human-induced pluripotent stem-cell-derived neurospheres promote motor functional recovery after spinal cord injury in mice. *Proceedings of the National Academy of Sciences of the United States of America*.

[89] Mizuno H., Tobita M., Uysal A. C. (2012). Concise review: adipose-derived stem cells as a novel tool for future regenerative medicine. *Stem Cells*.

[90] Zuk P. A., Zhu M., Mizuno H. (2001). Multilineage cells from human adipose tissue: implications for cell-based therapies. *Tissue Engineering*.

[91] Kokai L. E., Marra K., Rubin J. P. (2014). Adipose stem cells: biology and clinical applications for tissue repair and regeneration. *Translational Research*.

[92] Faroni A. A., Rothwell S. W., Grolla A. A., Terenghi G. G., Magnaghi V. V., Verkhratsky A. A. (2013). Differentiation of adipose-derived stem cells into Schwann cell phenotype induces expression of P2X receptors that control cell death. *Cell Death and Disease*.

[93] di Summa P. G., Kingham P. J., Raffoul W., Wiberg M., Terenghi G., Kalbermatten D. F. (2010). Adipose-derived stem cells enhance peripheral nerve regeneration. *Journal of Plastic, Reconstructive & Aesthetic Surgery*.

[94] Erba P., Mantovani C., Kalbermatten D. F., Pierer G., Terenghi G., Kingham P. J. (2010). Regeneration potential and survival of transplanted undifferentiated adipose tissue-derived stem cells in peripheral nerve conduits. *Journal of Plastic, Reconstructive & Aesthetic Surgery*.

[95] Santiago L. Y., Clavijo-Alvarez J., Brayfield C., Rubin J. P., Marra K. G. (2009). Delivery of adipose-derived precursor cells for peripheral nerve repair. *Cell Transplantation*.

[96] Widgerow A. D., Salibian A. A., Lalezari S., Evans G. R. D. (2013). Neuromodulatory nerve regeneration: adipose tissue-derived stem cells and neurotrophic mediation in peripheral nerve regeneration. *Journal of Neuroscience Research*.

[97] Kingham P. J., Kolar M. K., Novikova L. N., Novikov L. N., Wiberg M. (2014). Stimulating the neurotrophic and angiogenic properties of human adipose-derived stem cells enhances nerve repair. *Stem Cells and Development*.

[98] Tomita K., Madura T., Sakai Y., Yano K., Terenghi G., Hosokawa K. (2013). Glial differentiation of human adipose-derived stem cells: implications for cell-based transplantation therapy. *Neuroscience*.

[99] Georgiou M., Golding J. P., Loughlin A. J., Kingham P. J., Phillips J. B. (2015). Engineered neural tissue with aligned, differentiated adipose-derived stem cells promotes peripheral nerve regeneration across a critical sized defect in rat sciatic nerve. *Biomaterials*.

[100] Ilancheran S., Moodley Y., Manuelpillai U. (2009). Human fetal membranes: a source of stem cells for tissue regeneration and repair?. *Placenta*.

[101] Fairbairn N. G., Meppelink A. M., Ng-Glazier J. (2015). Augmenting peripheral nerve regeneration using stem cells: a review of current opinion. *World Journal of Stem Cells*.

[102] Matsuse D., Kitada M., Kohama M. (2010). Human umbilical cord-derived mesenchymal stromal cells differentiate into functional schwann cells that sustain peripheral nerve regeneration. *Journal of Neuropathology and Experimental Neurology*.

[103] Kuroda Y., Kitada M., Wakao S., Dezawa M. (2011). Mesenchymal stem cells and umbilical cord as sources for schwann cell differentiation: their potential in peripheral nerve repair. *Open Tissue Engineering and Regenerative Medicine Journal*.

[104] Pereira T., Gärtner A., Amorim I. (2014). Promoting nerve regeneration in a neurotmesis rat model using poly(DL-lactide-*ε*-caprolactone) membranes and mesenchymal stem cells from the Wharton's jelly: in vitro and in vivo analysis. *BioMed Research International*.

[105] Peng J., Wang Y., Zhang L. (2011). Human umbilical cord Wharton's jelly-derived mesenchymal stem cells differentiate into a Schwann-cell phenotype and promote neurite outgrowth in vitro. *Brain Research Bulletin*.

[106] Pan H.-C., Cheng F.-C., Chen C.-J. (2007). Post-injury regeneration in rat sciatic nerve facilitated by neurotrophic factors secreted by amniotic fluid mesenchymal stem cells. *Journal of Clinical Neuroscience*.

[107] Li Y., Guo L., Ahn H. S., Kim M. H., Kim S.-W. (2014). Amniotic mesenchymal stem cells display neurovascular tropism and aid in the recovery of injured peripheral nerves. *Journal of Cellular and Molecular Medicine*.

[108] Cogle C. R., Guthrie S. M., Sanders R. C., Allen W. L., Scott E. W., Petersen B. E. (2003). An overview of stem cell research and regulatory issues. *Mayo Clinic Proceedings*.

[109] Fischbach G. D., Fischbach R. L. (2004). Stem cells: science, policy, and ethics. *The Journal of Clinical Investigation*.

[110] Walsh S., Midha R. (2009). Practical considerations concerning the use of stem cells for peripheral nerve repair. *Neurosurgical focus*.

[111] Berger A. C., Beachy S. H., Olson S. (2014). *Stem Cell Therapies: Opportunities for Ensuring the Quality and Safety of Clinical Offerings: Summary of a Joint Workshop*.

[112] Palomo A. B. A., McLenachan S., Chen F. K. (2015). Prospects for clinical use of reprogrammed cells for autologous treatment of macular degeneration. *Fibrogenesis & Tissue Repair*.

[113] European Medicines Agency (2007). Regulation (EC) no 1394/2007 of the European parliament and of the council 324/121. *Official Journal of the European Union*.

[114] Kawakami M., Sipp D., Kato K. (2010). Regulatory impacts on stem cell research in Japan. *Cell Stem Cell*.

[115] Hara A., Sato D., Sahara Y. (2014). New governmental regulatory system for stem cell–based therapies in Japan. *Therapeutic Innovation & Regulatory Science*.

[116] United States Food and Drug Administration http://www.fda.gov/forpatients/approvals/fast/ucm20041766.htm.

[117] Knoepfler P. S. (2015). From bench to FDA to bedside: US regulatory trends for new stem cell therapies. *Advanced Drug Delivery Reviews*.

[118] Caplan A. I., West M. D. (2014). Progressive approval: a proposal for a new regulatory pathway for regenerative medicine. *Stem Cells Translational Medicine*.

[119] European Medicines Agency Adaptive pathways. http://www.ema.europa.eu/ema/index.jsp?curl=pages/regulation/general/general_content_000601.jsp.

[120] Culme-Seymour E. J., Mason C. (2012). ‘The little purple book’, 2nd edition: cell therapy and regenerative medicine glossary. *Regenerative Medicine*.

[121] Georgiou M., Bunting S. C. J., Davies H. A., Loughlin A. J., Golding J. P., Phillips J. B. (2013). Engineered neural tissue for peripheral nerve repair. *Biomaterials*.

[122] Unger C., Skottman H., Blomberg P., Sirac dilber M., Hovatta O. (2008). Good manufacturing practice and clinical-grade human embryonic stem cell lines. *Human Molecular Genetics*.

[123] Bieback K., Kinzebach S., Karagianni M. (2010). Translating research into clinical scale manufacturing of mesenchymal stromal cells. *Stem Cells International*.

[124] Bieback K., Wuchter P., Besser D. (2012). Mesenchymal stromal cells (MSCs): science and f(r)iction. *Journal of Molecular Medicine*.

[125] Arcidiacono J. A., Blair J. W., Benton K. A. (2012). US food and drug administration international collaborations for cellular therapy product regulation. *Stem Cell Research & Therapy*.

[126] Campbell A., Nycum G. (2005). Harmonizing the international regulation of embryonic stem cell research: possibilities, promises and potential pitfalls. *Medical Law International*.

[127] Lindvall O., Hyun I., Ärhlund-Richter L. (2008). *Guidelines for the Clinical Translation of Stem Cells*.

[128] Pirnay J.-P., Vanderkelen A., De Vos D. (2013). Business oriented EU human cell and tissue product legislation will adversely impact member states' health care systems. *Cell and Tissue Banking*.

[129] Human Tissue Authority https://www.hta.gov.uk/policies/regulating-human-embryonic-stem-cell-lines-human-application.

[130] Human Tissue

[131] Beyer Nardi N., da Silva L. (2006). Mesenchymal stem cells: isolation, in vitro expansion and characterization. *Handbook of Experimental Pharmacology*.

[132] Soleimani M., Nadri S. (2009). A protocol for isolation and culture of mesenchymal stem cells from mouse bone marrow. *Nature Protocols*.

[133] Francis M. P., Sachs P. C., Elmore L. W., Holt S. E. (2010). Isolating adipose-derived mesenchymal stem cells from lipoaspirate blood and saline fraction. *Organogenesis*.

[134] Can A., Balci D., Vemuri M., Chase L. G., Rao M. S. (2011). Isolation, culture, and characterization of human umbilical cord stroma-derived mesenchymal stem cells. *Mesenchymal Stem Cell Assays and Applications*.

[135] Byun J., Kang E. J., Park S. (2012). Isolation of human mesenchymal stem cells from the skin and their neurogenic differentiation in vitro. *Journal of the Korean Association of Oral and Maxillofacial Surgeons*.

[136] Butler M. G., Menitove J. E. (2011). Umbilical cord blood banking: an update. *Journal of Assisted Reproduction and Genetics*.

[137] Kim D.-W., Staples M., Shinozuka K., Pantcheva P., Kang S.-D., Borlongan C. V. (2013). Wharton's jelly-derived mesenchymal stem cells: phenotypic characterization and Optimizing their therapeutic potential for clinical applications. *International Journal of Molecular Sciences*.

[138] Kim E. Y., Lee K.-B., Kim M. K. (2014). The potential of mesenchymal stem cells derived from amniotic membrane and amniotic fluid for neuronal regenerative therapy. *BMB Reports*.

[139] Zhu Y., Yang Y., Zhang Y. (2014). Placental mesenchymal stem cells of fetal and maternal origins demonstrate different therapeutic potentials. *Stem Cell Research & Therapy*.

[140] Hjortholm N., Jaddini E., Hałaburda K., Snarski E. (2013). Strategies of pain reduction during the bone marrow biopsy. *Annals of Hematology*.

[141] Riley R. S., Hogan T. F., Pavot D. R. (2004). A pathologist's perspective on bone marrow aspiration and biopsy: I. Performing a bone marrow examination. *Journal of Clinical Laboratory Analysis*.

[142] Strioga M., Viswanathan S., Darinskas A., Slaby O., Michalek J. (2012). Same or not the same? comparison of adipose tissue-derived versus bone marrow-derived mesenchymal stem and stromal cells. *Stem Cells and Development*.

[143] Stolzing A., Jones E., McGonagle D., Scutt A. (2008). Age-related changes in human bone marrow-derived mesenchymal stem cells: consequences for cell therapies. *Mechanisms of Ageing and Development*.

[144] Fraser J. K., Wulur I., Alfonso Z., Hedrick M. H. (2006). Fat tissue: an underappreciated source of stem cells for biotechnology. *Trends in Biotechnology*.

[145] Pisciotta A., Carnevale G., Meloni S. (2015). Human Dental pulp stem cells (hDPSCs): isolation, enrichment and comparative differentiation of two sub-populations Integrative control of development. *BMC Developmental Biology*.

[146] Martens W., Bronckaers A., Politis C., Jacobs R., Lambrichts I. (2013). Dental stem cells and their promising role in neural regeneration: an update. *Clinical Oral Investigations*.

[147] Guo W., Patzlaff N. E., Jobe E. M., Zhao X. (2012). Isolation of multipotent neural stem or progenitor cells from both the dentate gyrus and subventricular zone of a single adult mouse. *Nature Protocols*.

[148] Jakel R. J., Schneider B. L., Svendsen C. N. (2004). Using human neural stem cells to model neurological disease. *Nature Reviews Genetics*.

[149] Gu S., Shen Y., Xu W. (2010). Application of fetal neural stem cells transplantation in delaying denervated muscle atrophy in rats with peripheral nerve injury. *Microsurgery*.

[150] Ilic D., Polak J. M. (2011). Stem cells in regenerative medicine: introduction. *British Medical Bulletin*.

[151] Amoh Y., Hamada Y., Aki R., Kawahara K., Hoffman R. M., Katsuoka K. (2010). Direct transplantation of uncultured hair-follicle Pluripotent Stem (hfPS) cells promotes the recovery of peripheral nerve injury. *Journal of Cellular Biochemistry*.

[152] Mimura S., Kimura N., Hirata M. (2011). Growth factor-defined culture medium for human mesenchymal stem cells. *International Journal of Developmental Biology*.

[153] Sverdlov E. D., Mineev K. (2013). Mutation rate in stem cells: an underestimated barrier on the way to therapy. *Trends in Molecular Medicine*.

[154] Wang Y., Zhang Z., Chi Y. (2013). Long-term cultured mesenchymal stem cells frequently develop genomic mutations but do not undergo malignant transformation. *Cell Death & Disease*.

[155] Peterson S. E., Loring J. F. (2014). Genomic instability in pluripotent stem cells: implications for clinical applications. *The Journal of Biological Chemistry*.

[156] Oliveira J. T., Almeida F. M., Biancalana A. (2010). Mesenchymal stem cells in a polycaprolactone conduit enhance median-nerve regeneration, prevent decrease of creatine phosphokinase levels in muscle, and improve functional recovery in mice. *Neuroscience*.

[157] Tan C. W., Ng M., Ohnmar H. (2013). Sciatic nerve repair with tissue engineered nerve: olfactory ensheathing cells seeded poly(lactic-co-glygolic acid) conduit in an animal model. *Indian Journal of Orthopaedics*.

[158] Jung S., Panchalingam K. M., Rosenberg L., Behie L. A. (2012). Ex vivo expansion of human mesenchymal stem cells in defined serum-free media. *Stem Cells International*.

[159] Horwitz E. M., Gordon P. L., Koo W. K. K. (2002). Isolated allogeneic bone marrow-derived mesenchymal cells engraft and stimulate growth in children with osteogenesis imperfecta: implications for cell therapy of bone. *Proceedings of the National Academy of Sciences of the United States of America*.

[160] Sundin M., Ringdén O., Sundberg B., Nava S., Götherström C., Le Blanc K. (2007). No alloantibodies against mesenchymal stromal cells, but presence of anti-fetal calf serum antibodies, after transplantation in allogeneic hematopoietic stem cell recipients. *Haematologica*.

[161] Chase L. G., Lakshmipathy U., Solchaga L. A., Rao M. S., Vemuri M. C. (2010). A novel serum-free medium for the expansion of human mesenchymal stem cells. *Stem Cell Research & Therapy*.

[162] Escobedo-Lucea C., Bellver C., Gandia C. (2013). A xenogeneic-free protocol for isolation and expansion of human adipose stem cells for clinical uses. *PLoS ONE*.

[163] Usta S. N., Scharer C. D., Xu J., Frey T. K., Nash R. J. (2014). Chemically defined serum-free and xeno-free media for multiple cell lineages. *Annals of Translational Medicine*.

[164] Rojewski M. T., Fekete N., Baila S. (2013). GMP-compliant isolation and expansion of bone marrow-derived MSCs in the closed, automated device quantum cell expansion system. *Cell Transplantation*.

[165] Trokovic R., Weltner J., Noisa P., Raivio T., Otonkoski T. (2015). Combined negative effect of donor age and time in culture on the reprogramming efficiency into induced pluripotent stem cells. *Stem Cell Research*.

[166] Bressan E., Ferroni L., Gardin C. (2012). Donor age-related biological properties of human dental pulp stem cells change in nanostructured scaffolds. *PLoS ONE*.

[167] Lee D.-H., Ng J., Kim S.-B., Sonn C. H., Lee K.-M., Han S.-B. (2015). Effect of donor age on the proportion of mesenchymal stem cells derived from anterior cruciate ligaments. *PLoS ONE*.

[168] Siegel G., Kluba T., Hermanutz-Klein U., Bieback K., Northoff H., Schäfer R. (2013). Phenotype, donor age and gender affect function of human bone marrow-derived mesenchymal stromal cells. *BMC Medicine*.

[169] Choudhery M. S., Badowski M., Muise A., Pierce J., Harris D. T. (2014). Donor age negatively impacts adipose tissue-derived mesenchymal stem cell expansion and differentiation. *Journal of Translational Medicine*.

[170] Choi J.-S., Lee B.-J., Park H.-Y. (2015). Effects of donor age, long-term passage culture, and cryopreservation on tonsil-derived mesenchymal stem cells. *Cellular Physiology and Biochemistry*.

[171] Rai B., Lin J. L., Lim Z. X. H., Guldberg R. E., Hutmacher D. W., Cool S. M. (2010). Differences between in vitro viability and differentiation and in vivo bone-forming efficacy of human mesenchymal stem cells cultured on PCL-TCP scaffolds. *Biomaterials*.

[172] Burns J. S., Rasmussen P. L., Larsen K. H., Schrøder H. D., Kassem M. (2010). Parameters in three-dimensional osteospheroids of telomerized human mesenchymal (Stromal) stem cells grown on osteoconductive scaffolds that predict in vivo bone-forming potential. *Tissue Engineering Part: A*.

[173] Fossett E., Khan W. S. (2012). Optimising human mesenchymal stem cell numbers for clinical application: a literature review. *Stem Cells International*.

[174] Zhou J.-M., Xing F.-Y., Shi J.-J., Fang Z.-F., Chen X.-J., Chen F. (2008). Quality of embryonic bodies and seeding density effects on neural differentiation of mouse embryonic stem cells. *Cell Biology International*.

[175] Hunt C. J. (2011). Cryopreservation of human stem cells for clinical application: a review. *Transfusion Medicine and Hemotherapy*.

[176] Heng B. C., Kuleshova L. L., Bested S. M., Liu H., Cao T. (2005). The cryopreservation of human embryonic stem cells. *Biotechnology and Applied Biochemistry*.

[177] Lee J. E., Lee D. R. (2011). Human embryonic stem cells: derivation, maintenance and cryopreservation. *International Journal of Stem Cells*.

[178] Loutradi K. E., Kolibianakis E. M., Venetis C. A. (2008). Cryopreservation of human embryos by vitrification or slow freezing: a systematic review and meta-analysis. *Fertility and Sterility*.

[179] Holm F., Ström S., Inzunza J. (2010). An effective serum-and xeno-free chemically defined freezing procedure for human embryonic and induced pluripotent stem cells. *Human Reproduction*.

[180] Lin P.-Y., Yang Y.-C., Hung S.-H. (2013). Cryopreservation of human embryonic stem cells by a programmed freezer with an oscillating magnetic field. *Cryobiology*.

[181] Bruder S. P., Jaiswal N., Haynesworth S. E. (1997). Growth kinetics, self-renewal, and the osteogenic potential of purified human mesenchymal stem cells during extensive subcultivation and following cryopreservation. *Journal of Cellular Biochemistry*.

[182] Ginis I., Grinblat B., Shirvan M. H. (2012). Evaluation of bone marrow-derived mesenchymal stem cells after cryopreservation and hypothermic storage in clinically safe medium. *Tissue Engineering—Part C: Methods*.

[183] Naaldijk Y., Staude M., Fedorova V., Stolzing A. (2012). Effect of different freezing rates during cryopreservation of rat mesenchymal stem cells using combinations of hydroxyethyl starch and dimethylsulfoxide. *BMC Biotechnology*.

[184] Hanna J., Hubel A. (2009). Preservation of stem cells. *Organogenesis*.

[185] Hsueh Y.-Y., Chang Y.-J., Huang T.-C. (2014). Functional recoveries of sciatic nerve regeneration by combining chitosan-coated conduit and neurosphere cells induced from adipose-derived stem cells. *Biomaterials*.

[186] Scholz T., Sumarto A., Krichevsky A., Evans G. R. D. (2011). Neuronal differentiation of human adipose tissue-derived stem cells for peripheral nerve regeneration in vivo. *Archives of Surgery*.

[187] Carriel V., Garrido-Gómez J., Hernández-Cortés P. (2013). Combination of fibrin-agarose hydrogels and adipose-derived mesenchymal stem cells for peripheral nerve regeneration. *Journal of Neural Engineering*.

[188] Suganuma S., Tada K., Hayashi K. (2013). Uncultured adipose-derived regenerative cells promote peripheral nerve regeneration. *Journal of Orthopaedic Science*.

[189] Orbay H., Uysal A. C., Hyakusoku H., Mizuno H. (2012). Differentiated and undifferentiated adipose-derived stem cells improve function in rats with peripheral nerve gaps. *Journal of Plastic, Reconstructive & Aesthetic Surgery*.

[190] Liu G.-B., Cheng Y.-X., Feng Y.-K. (2011). Adipose-derived stem cells promote peripheral nerve repair. *Archives of Medical Science*.

[191] Reid A. J., Sun M., Wiberg M., Downes S., Terenghi G., Kingham P. J. (2011). Nerve repair with adipose-derived stem cells protects dorsal root ganglia neurons from apoptosis. *Neuroscience*.

[192] Shen C.-C., Yang Y.-C., Liu B.-S. (2012). Peripheral nerve repair of transplanted undifferentiated adipose tissue-derived stem cells in a biodegradable reinforced nerve conduit. *Journal of Biomedical Materials Research Part: A*.

[193] Sun F., Zhou K., Mi W.-J., Qiu J.-H. (2011). Combined use of decellularized allogeneic artery conduits with autologous transdifferentiated adipose-derived stem cells for facial nerve regeneration in rats. *Biomaterials*.

[194] Wei Y., Gong K., Zheng Z. (2011). Chitosan/silk fibroin-based tissue-engineered graft seeded with adipose-derived stem cells enhances nerve regeneration in a rat model. *Journal of Materials Science: Materials in Medicine*.

[195] Zhang Y., Luo H., Zhang Z. (2010). A nerve graft constructed with xenogeneic acellular nerve matrix and autologous adipose-derived mesenchymal stem cells. *Biomaterials*.

[196] Ghoreishian M., Rezaei M., Beni B. H., Javanmard S. H., Attar B. M., Zalzali H. (2013). Facial nerve repair with gore-tex tube and adipose-derived stem cells: An animal study in dogs. *Journal of Oral and Maxillofacial Surgery*.

[197] Sakar M., Korkusuz P., Demirbilek M. (2014). The effect of poly(3-hydroxybutyrate-co-3-hydroxyhexanoate) (PHBHHx) and human mesenchymal stem cell (hMSC) on axonal regeneration in experimental sciatic nerve damage. *International Journal of Neuroscience*.

[198] Mohammadi R., Azizi S., Delirezh N., Hobbenaghi R., Amini K., Malekkhetabi P. (2012). The use of undifferentiated bone marrow stromal cells for sciatic nerve regeneration in rats. *International Journal of Oral and Maxillofacial Surgery*.

[199] Matsushita K., Wang W., Itoh S., Domon T., Funahashi M., Totsuka Y. (2012). Dental pulp can be a good candidate for nerve grafting in a xeno-graft model. *Journal of Neuroscience Methods*.

